# Lin28a Protects against Hypoxia/Reoxygenation Induced Cardiomyocytes Apoptosis by Alleviating Mitochondrial Dysfunction under High Glucose/High Fat Conditions

**DOI:** 10.1371/journal.pone.0110580

**Published:** 2014-10-14

**Authors:** Mingming Zhang, Xiaolin Niu, Jianqiang Hu, Yuan Yuan, Shuhong Sun, Jiaxing Wang, Wenjun Yu, Chen Wang, Dongdong Sun, Haichang Wang

**Affiliations:** 1 Department of Cardiology, Xijing Hospital, Fourth Military Medical University, Xi’an, China; 2 Department of Cardiology, Tangdu Hospital, Fourth Military Medical University, Xi’an, China; 3 Department of Cardiology, Corps Hospital, Chinese People’s Armed Police Forces, Xi’an, China; National Institutes of Health, United States of America

## Abstract

**Aim:**

The aim of the present study was to investigate the role of Lin28a in protecting against hypoxia/reoxygenation (H/R)-induced cardiomyocytes apoptosis under high glucose/high fat (HG/HF) conditions.

**Methods:**

Primary cardiomyocytes which were isolated from neonatal mouse were randomized to be treated with lentivirus carrying Lin28a siRNA, Lin28acDNA 72 h before H/R (9 h/2 h). Cardiomyocytes biomarkers release (LDH and CK), cardiomyocytes apoptosis, mitochondria biogenesis and morphology, intracellular reactive oxygen species (ROS) production, ATP content and inflammatory cytokines levels after H/R injury in high glucose/high fat conditions were compared between groups. The target proteins of Lin28a were examined by western blot analysis.

**Results:**

Our results revealed that Lin28a cDNA transfection (overexpression) significantly inhibited cardiomyocyte apoptotic index, improved mitochondria biogenesis, increased ATP production and reduced ROS production as compared with the H/R group in HG/HF conditions. Lin28a siRNA transfection (knockdown) rendered the cardiomyocytes more susceptible to H/R injury as evidenced by increased apoptotic index, impaired mitochondrial biogenesis, decreased ATP production and increased ROS level. Interestingly, these effects of Lin28a were blocked by pretreatment with the PI3K inhibitor wortmannin. Lin28a overexpression increased, while Lin28a knockdown inhibited IGF1R, Nrf-1, Tfam, p-IRS-1, p-Akt, p-mTOR, p-p70s6k, p-AMPK expression levels after H/R injury in HG/HF conditions. Moreover, pretreatment with wortmannin abolished the effects of Lin28a on the expression levels of p-AKT, p-mTOR, p-p70s6k, p-AMPK.

**Conclusions:**

The present results suggest that Lin28a inhibits cardiomyocytes apoptosis by enhancing mitochondrial biogenesis and function under high glucose/high fat conditions. The mechanism responsible for the effects of Lin28a is associated with the PI3K/Akt dependent pathway.

## Introduction

The escalating epidemic of diabetes (DM) represents one of the most pressing and costly biomedical challenges confronting modern society [1]. This growth in diabetes prevalence is occurring in both developing and developed countries. The World Health Organization (WHO) predicts the number of diabetic patients will increase to at least 366 million by the year 2030 [2]. Cardiovascular diseases (CVD) are the most prevalent cause of mortality and morbidity among diabetic patients [3]–[5]. Although the efficacy of glycemic control and other cardiovascular risk factors in diabetic patients have been demonstrated, the majority of diabetic patients never achieve the goals established by guidelines issued by diabetes societies [6]–[8].

Mitochondrial biogenesis is a dynamically regulated process where mitochondrial maintenance, quantity, and activity constantly adapt to the cell’s changing bioenergetic needs. Due to the constant demand for high ATP levels for maintaining contractile activity in the heart, the loss of mitochondrial number and function can have an extremely adverse impact on cardiac muscle. In addition, mitochondria are involved in many other cellular processes including cell death, and are regarded as a major target of MI/R injury [9], [10].

Lin28a has been used to reprogram human somatic cells into induced pluripotent stem (iPS) cells [11]. Recent data are beginning to uncover roles for Lin28a in glucose metabolism. Zhu et al reported that overexpression of murine Lin28a promoted an insulin-sensitized state that resisting diabetes in mice, while overexpression of let-7 resulted in insulin resistance and impaired glucose tolerance [12]. Glucose tolerance tests (GTT) and insulin tolerance tests (ITT) also demonstrated that muscle specific loss of Lin28a led to insulin resistance and impaired glucose uptake [13].

The underlying mechanisms which cause increased cardiovascular risks in patients with diabetes are poorly understood. Our previous data demonstrated that diabetes aggravates cardiac ischemia/reperfusion injury as evidenced by decreased left ventricular ejection fraction, increased cardiomyocyte apoptosis and microvascular endothelial barrier dysfunction [14], [15]. According to our recent preliminary data, both diabetes and ischemic insult lead to decreased expression of Lin28a, increased cardimyocyte apoptosis and impaired mitochondrial function. Nevertheless, little is known about the role and mechanisms of Lin28a protecting against cardiac I/R injury in diabetic mice. In the present study, we isolated primary cardiomyocytes from neonatal mouse and established an in vitro model of hypoxia/reoxygenation (H/R) which resembles I/R in vivo. High glucose and high fat culture medium was used to mimic diabetes in vivo. The effects of Lin28a overexpression or knockdown on cardiomyocyte apoptosis, mitochondrial biogenesis and function after hypoxia/reoxygenation injury under high glucose/high fat conditions were investigated.

## Materials and Methods

### Cell culture

Primary cultures of cardiomyocytes were isolated from ventricle of neonatal C57BL/6 mice (1–3 days) as described previously [16]. Neonatal C57BL/6 mice received humane care in Adherence with the National Institutes of Health Guidelines on the Use of Laboratory Animals and were approved by the Fourth Military Medical University Committee on Animal Care (ID:20130722). The neonatal mice (1–3 day old) were disinfected with 75% ethanol and then killed by decapitation. The chest was opened and the heart was rapidly removed and placed in the cold PBS solution. Myocardium specimen was cut in small pieces and washed, followed by digestion steps with collagenase type 2. After that, the cell suspension was centrifuged (800 g for 5 min). The supernatant was then removed and the cell pellet was resuspended in medium supplemented with 10% fetal bovine serum. These steps were repeated until the tissue fragments had disappeared. The dissociated cells were replated in a culture flask at 37°C for 1 h to enrich the culture with cardiomyocytes. The non-adherent cardiomyocytes were collected and then were plated onto gelatin-coated plates. The preparation was carried out at 37°C, in the presence of 95% O_2_ and 5% CO_2_. Neonatal mouse ventricular myocytes culture media contains glucose (25 mmol/L) [17] and saturated FFA palmitate (16∶0; 500 umol/L) [18]. After 48 h, Lin28a-silencing lentivirus and Lin28a-cDNA lentivirus were transfected into cardiomyocytes. After 72 h transfection, the cells were subjected to H/R injury. Cardiomyocytes H/R injury model was constructed as previously described [15]. For induction of H/R injury, cells were cultured in D-Hanks solution in a modular incubator chamber (BioSpherix) with 1% O_2_, 5% CO_2_ and 94% N2 for 9 h (hypoxia for 9 h), then exposed to atmosphere of 21% O_2_, 5% CO_2_ and 74% N2 and cultured with HG/HF for 2 h (reoxygenation for 2 h). Cardiomyocytes were randomly divided into the following groups:(i) Control (CON); (ii) High glucose/High fat (HG/HF); (iii) Hypoxia/Reoxygenation (H/R)+HG/HF (H/R); (iv) HG/HF+siControl+H/R (H/R+siControl); (v) HG/HF+Lin28asiRNA+H/R (H/R+siLin28a); (vi) HG/HF+Control vector+H/R (H/R+Control vector); (vii) HG/HF+Lin28a Overexpression+H/R (H/R+Lin28a); (viii) HG/HF+Lin28aOverexpression+Wortmannin+H/R (H/R+Lin28a+W). Wortmannin (a specific PI3K inhibitor, 5×10^−6^ mol/L) was added in HG/HF media 24 h before H/R in the H/R+Lin28a+W group.

### Quantitative real-time PCR (qRT-PCR) analysis

Cardiomyocytes were harvested, and total RNA was isolated by using Trizol reagents (Invitrogen). The first strand cDNA was generated from total RNA with reverse transcriptase (TAKARA, JAPAN) and used as the template for qRT-PCR analysis. GAPDH cDNA and U6 were used as an internal control to normalize variances. Primers used were as follows: Lin28a, 50- GAGGCAGTGGAGTTCACCTTTA- 30 (forward) and 50- TCCTTGGCATGGTGGTCTA-30 (reverse); GAPDH, 50-GGCACAGTCAAGGCTGAGAATG-30 (forward) and 50-ATGGTGGTG AAGACGCCAGTA-30 (reverse); let7a, 50- CGGTGAGGTAGTAGGTTGTATAGTT- 30 (forward). The expression of mature miRNAs was assayed using poly (A)-tail RT followed by quantitative real-time PCR analysis. All reagents for the poly (A)-tail RT were obtained from SYBR PrimeScriptmiRNA RT-PCR kit TaKaRa (Code No. RR716). The mRNAs RT-PCR was performed using the RT-PCR System from TaKaRa (Cat.#RR047A). RT was performed in a GeneAmp PCR system 2400 Thermal Cycler (Perkin-Elmer, Norwalk CT, USA). PCR conditions were 30 s at 94°C, 30 s at 58°C, and 30 s at 72°C. The PCR products were detected by using real-time PCR detection kit (RR716, TAKARA) following the handbook protocols in ABI 7500 sequence detection system. The results were analyzed by calculating the Ct values for target genes in the samples. The 2^−ΔΔCT^ method was used to calculate the relative expression levels of each gene. ΔCT value = Ct value- internal reference Ct value. ΔΔCT value = ΔCT value -CON group ΔCT value. The 2^−ΔΔCT^ value was the final value of the gene expression levels.

### Lentiviral-vectored Lin28a siRNA and Lin28a cDNA

Lentivirus carrying Lin28a siRNA or Lin28a cDNA was purchased from Gene-Pharma Company (Shanghai, China). The RNAi sequence targeting mouse Lin28a is 50-GCAGTGGAGTTCACCTTTAAG -30.

### Determination of Cardiomyocyte Apoptosis

Cardiomyocyte apoptosis was determined by terminal deoxyribonucleotidyl transferase- mediated dUTP-biotin nick end labeling (TUNEL) staining as previously described [23]. TUNEL staining was performed with fluorescein-dUTP (In Situ Cell Death Detection Kit; Roche Diagnostics) for apoptotic cell nuclei and 4, 6- diamidino −2 -phenylindole (DAPI) (Sigma) stained all cell nuclei. AI is the number of TUNEL-positive cardiomyocytes divided by the total number of cardiomyocytes stained with DAPI from a total of 10 fields per group. Cleaved Caspase-3, Caspase-3, Bcl-2, Bax were detected by Western Blot evaluation. Flow cytometric analysis of cellular apoptosis was performed as previously described [16]. In brief, cardiomyocytes were harvested and stained with annexin V (Invitrogen) and propidium iodide (PI). Data acquisition and analysis were performed using a flow cytometer (FACSort- B0008; Becton- Dickinson, Franklin Lakes, NJ, USA) and Cell Quest Pro software, respectively. All of these assays were performed in a blinded manner.

### Transmission electron microscopy (TEM)

The cells were harvested using 0.25% trypsin, then transfered into 1.5 mL Eppendorf tubes and centrifuged at 180×g for 15 min. As described in literature [19], glutaraldehyde (2.5%) was used for immobilizing the cells at 4°C for 2 h, then rinsed in buffer, postfixed in 1% osmium tetroxide in 0.1 M potassium phosphate for 2 h at 4°C, dehydrated in ethanol and embedded in Epon-Araldite resin. Then ultrathin sections with the thickness of 75 nm were stained with uranium acetate and lead citrate and images were observed under a JEM-2000EX transmission electron microscope at 60 kV.

### ATP content Determination

The ATP content of neonatal mouse ventricular myocytes was measured using an ATP bioluminescent assay kit (Beyotime, China).

### Determining citrate synthase (CS) activity

CS activity of neonatal mouse ventricular myocytes was measured using a commercially available citrate synthase activity assay kit (Sigma, USA).

### Assays for mitochondrial enzyme activities

Enzymatic activities of mitochondrial complexes I–V were measured as previously described [20]. The mitochondrial marker enzyme CS was used as a reference.

### Western Blot Evaluation

Total proteins from cardiomyocytes were separated by SDS-PAGE, blotted and probed with anti-β-actin antibody (Santa Cruz, CA, USA), anti-Lin28a (Abcam, Cambridge, MA, UK), anti-p-p70S6k (Thr389), anti-p70S6k, anti-phospho-Akt (ser473), anti-Akt, anti-mTOR, anti-phospho-mTOR (Ser2448) (Cell Signaling, Danvers, MA, USA), p-AMPK (Thr172), Adenine mononucleotide protein kinase (AMPK), Acetylated-Lysine, Peroxisome proliferator-activated 5 receptor-γ coactivator-1α (PGC-1α), nuclear respiratory factor 1 (Nrf-1), mitochondrial6 transcription factor A (Tfam) (Cell Signaling Technology, Beverly, MA, USA) anti-Cleaved Caspase-3, anti-Caspase-3, anti-Bcl-2, anti-Bax (Sigma, St Louis, MO, USA). The Bradford assay (Bio-Rad Laboratories, Hercules, CA, USA) was used to quantify protein concentrations. The blots were visualized with a chemiluminescence system (Amersham Bioscience, Buchinghamshire, UK). The signals were quantified by densitometry and normalized to β-actin.

### Assessment of Cardiomyocyte injury

Culture medium was collected to assess the lactate dehydrogenase (LDH) and creatine kinase (CK). Standard techniques using commercialized assay kits according to the manufacturer’s instructions (Nanjing Jiancheng Bioengineering Institute, China) were performed for analysis.

Values were expressed in international units (IU) per liter.

### Determination of Cardiomyocyte TNF-α and IL-6 Activity

Following the 2 h reoxygenation period, cellular supernatants were collected for cytokines activity analysis. The amount of TNF-α and IL-6 in the culture supernatant was determined using an ELISA method according to the manufacturers’ instructions. TNF-α and IL-6 ELISA kits were supplied by R&D (Minneapolis, MN, USA). Values are expressed as pictograms per milliliter supernatants.

### Determination of Cardiomyocyte ROS production

The superoxide sensitive fluorescent dye dihydroethidium (DHE) was used to evaluate *in* *situ* formation of O2^−^ as followed [21], [22]. Cardiomyocytes incubated with DHE (DHE, 1∶1000 dilution, Beyotime Institute of Biotechnology, Nanjing, China) at 37°C for 30 minutes. Cardiomyocytes were visualized with microscope. Images were collected and stored digitally.

### Measurement of malondialdehyde (MDA) and superoxide dismutase (SOD) activity

MDA and SOD activities in cardiomyocytes were measured using commercialized assay kits according to the manufacturer’s instructions (Nanjing Jiancheng Bioengineering Institute, China) and were used as indexes of lipid superoxide and oxygen free radical level in the cardiomyocytes.

### Statistical analysis

All values and figures were expressed as mean ± SD. Comparison between groups were subjected to ANOVA followed by Bonferroni correction for post hoc t-test. Data expressed as proportions were assessed with a Chi-square test. Two sided tests have been used throughout, and *P* values<0.05 were considered statistically significant. SPSS software package version 14.0 (SPSS, Chicago, IL) was used for data analysis.

## Results

### Lin28a overexpression inhibits let7a expression in cardiomyocytes after H/R injury under HG/HF conditions

**Figure 1:**
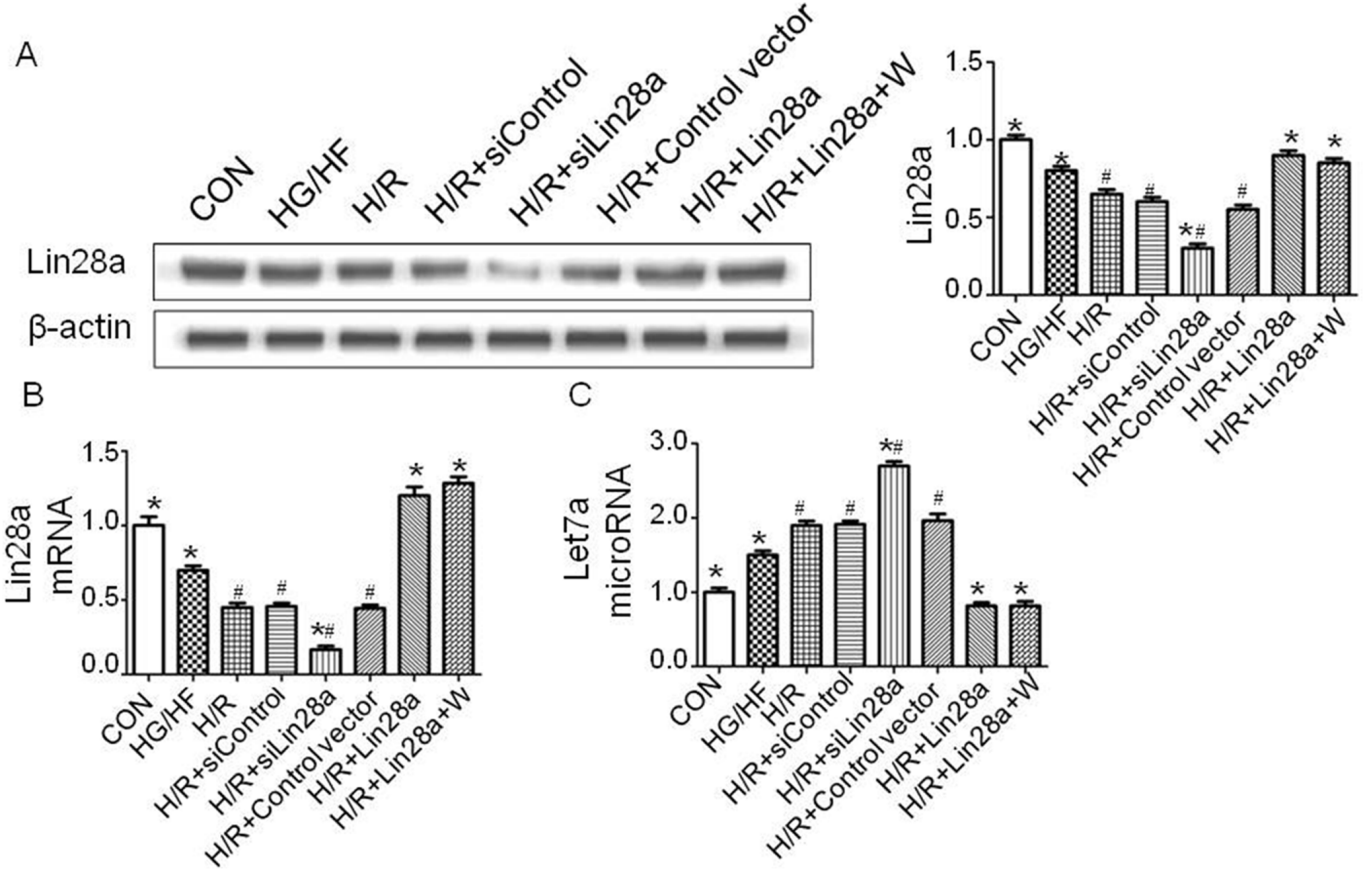
Validation of Lin28a overexpression and Lin28a knockdown. Lin28a expression levels with representative gel blots of Lin28a and β-actin (loading control) were shown (A); Lin28a mRNA expression as evaluated by real-time PCR analysis (B); Let7a (microRNA) expression as evaluated by real-time PCR analysis (C). Columns and bars represent mean ± SD. Data were obtained from at least three independent experiments. W, wortmannin.*p<0.05 vs. H/R, ^#^p<0.05 vs. H/R+Lin28a.

Primary cardiomyocytes were treated with lentivirus carrying Lin28a siRNA, Lin28a cDNA 72 h before H/R (9 h/2 h). HG/HF treatment decreased Lin28a expression levels compared with the control group. H/R group decreased Lin28a expression levels compared with the HG/HF group (Figure 1A, 1B). Lin28a overexpression inhibited, while Lin28a siRNA administration promoted let7a expression as indicated by real-time PCR analysis (Figure 1C). HG/HF treatment increased Let7a expression levels compared with the control group. H/R group increased Lin28a expression levels compared with the HG/HF group (Figure 1A, 1B). There was no differences between H/R+Lin28a group and H/R+Lin28a+W group in Lin28a and Let7a expression levels (Figure 1A–1C). The Ct values were presented in table S1.

### Lin28a overexpression inhibits cardiomyocytes apoptosis after H/R injury in HG/HF incubation

**Figure 2:**
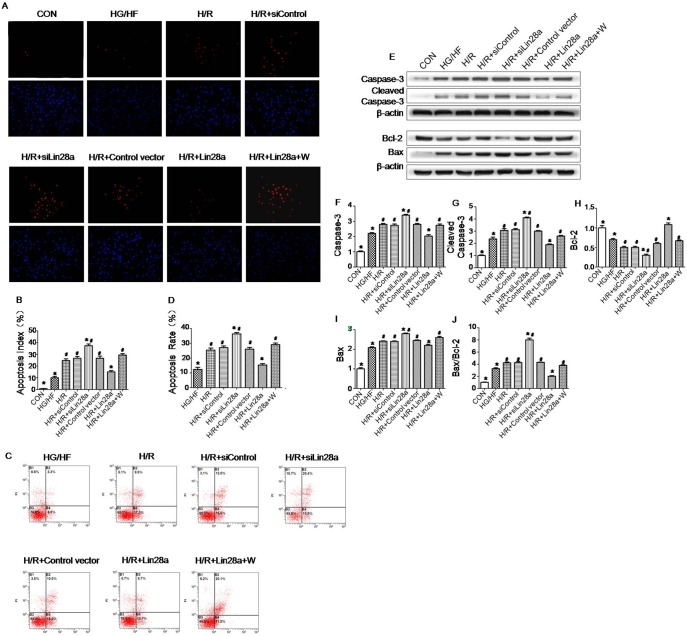
Antiapoptotic effect of Lin28a overexpression on cardiomyocytes after H/R injury in HG/HF conditions. Representative images of TUNEL-stained primary neonatal cardiomyocytes after H/R stress in HG/HF conditions (A). Apoptosis of the primary cardiomyocytes determined by Cy3-annexinV/PI double staining and flow cytometry (n = 3). Region B2: late apoptotic cells (Cy3/PI, where Cy3 is cyanine-3 and PI is propidium iodide); Region B3: vital cells; Region B4: early apoptotic cells (C). Apoptotic index is expressed as the percentage of TUNEL-positive myocytes (in red) over total nuclei determined by DAPI staining(B). Apoptotic rate is expressed as the percentage of late apoptotic cells and early apoptotic cells(D). Protein expression with representative gel blots of Caspase-3, Cleaved Caspase-3, Bcl-2, Bax and β-actin (loading control) (E). Caspase-3(F); Cleaved Caspase-3(G); Bcl-2(H); Bax(I); Bax/Bcl-2 ratio(J). The columns and error bars represent means and SD. Data were obtained from at least three independent experiments.*P<0.05 vs. H/R, ^#^P<0.05 vs. H/R+Lin28a.

As is shown in representative TUNEL images (Figure 2A), TUNEL-positive myocytes were less observed in Lin28a overexpression group as compared with the H/R group. Concomitantly, Cleaved Caspase-3, Caspase-3 and Bax protein levels evaluated by western blot were down-regulated by Lin28a overexpression. The Bcl-2 protein level evaluated by western blot was up-regulated by Lin28a overexpression. Furthermore, Lin28a overexpression decreased Bax/Bcl-2 ratio (Figure 2E–2J). Cardiomyocytes were likewise treated and analyzed by flow cytometry. The results also showed that Lin28a overexpression inhibited cellular apoptosis (Figure 2C). Lentivirus carrying Lin28a siRNA transduced cardiomyocytes showed a trend of cardiomyocytes.

H/R-aggravated apoptosis in HG/HF incubation, as demonstrated by enhanced expression levels of Cleaved Caspase-3, Caspase-3, Bax protein and Bax/Bcl-2 ratio (Figure 2E–2J), increased TUNEL (red) staining in cardiomyocytes (Figure 2A, 2B) and facilitated cellular apoptosis by flow cytometry analysis (Figure 2C, 2D). In addition, the anti-apoptotic effect of Lin28a overexpression on cardiomyocytes was abolished by wortmannin pretreatment as evidenced by enhanced expression levels of Cleaved Caspase-3, Caspase-3, Bax protein and Bax/Bcl-2 ratio (Figure 2E–2J), increased TUNEL (red) staining in cardiomyocytes (Figure 2A, 2B) and facilitated cellular apoptosis by flow cytometry analysis (Figure 2C, 2D) in comparison to the H/R+Lin28a group.

### Lin28a overexpression protects against cardiomyocytes ultrastructure impairment, enhances mitochondrial biogenesis after H/R injury under HG/HF conditions

**Figure 3:**
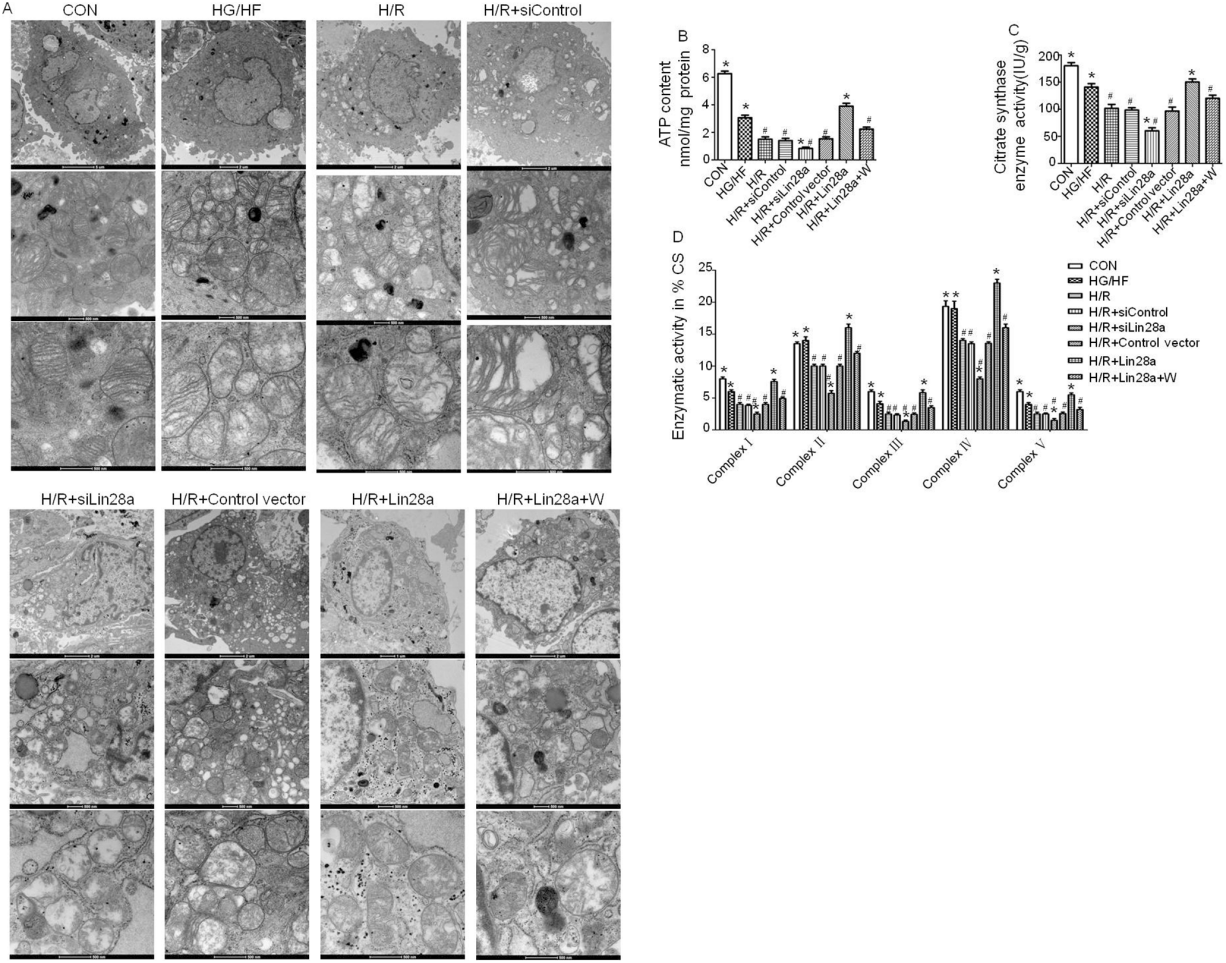
Cardiomyocytes ultrastructure changes as evaluated by transmission electron microscopy. Cardiomyocytes mitochondria size and the destruction of cristae (magnification: medial panel×20500; lower panel×43000) after H/R (9 h/2 h) injury in HG/HF (high glucose/high fat, 25 Mm glucose, and 500 uM sodium palmitate) conditions were shown (A). ATP content and citratesynthase activity in neonatal mouse ventricular myocytes (B, C). Enzymatic activities of complexes I–V were determined in mitochondria isolated from neonatal mouse ventricular myocytes (D). Columns and bars represent mean ± SD. Data were obtained from at least three independent experiments.*p<0.05 vs. H/R, #p<0.05 vs. H/R+Lin28a.

Electron microscope showed that cardiomyocytes mitochondria were found greater in size with the destruction of cristae after cardiomyocytes H/R (9 h/2 h) injury in HG/HF conditions. Lin28a overexpression alleviated mitochondria ultrastructure impairment with intact membrane although still with the destruction of cristae in part and this effect was abolished by wortmannin pretreatment. Lin28a siRNA administration aggravated mitochondria ultrastructure impairment compared with the H/R group (Figure 3A).

ATP content, CS activity and complexes I/III/V activities of the cardiomyocytes mitochondrial were remarkably decreased after HG/HF incubation compared with the control group (Figure 3B–3D). Lin28a overexpression increased, while Lin28a siRNA administration decreased the levels of ATP content, CS activity, and complexes I /II/III/IV/V activities compared with the H/R group (Figure 3B–3D). Interestingly, the effects of Lin28a on the levels of ATP content, CS activity, and complexes I/II/III/IV/V activities was abolished by wortmannin pretreatment (Figure 3B–3D).

### Lin28a overexpression increases IGF1R, Nrf-1, Tfam and p-IRS-1, p-Akt, p-mTOR, p-p70s6k, p-AMPK expression in cardiomyocytes exposed to H/R injury in HG/HF incubation

**Figure 4:**
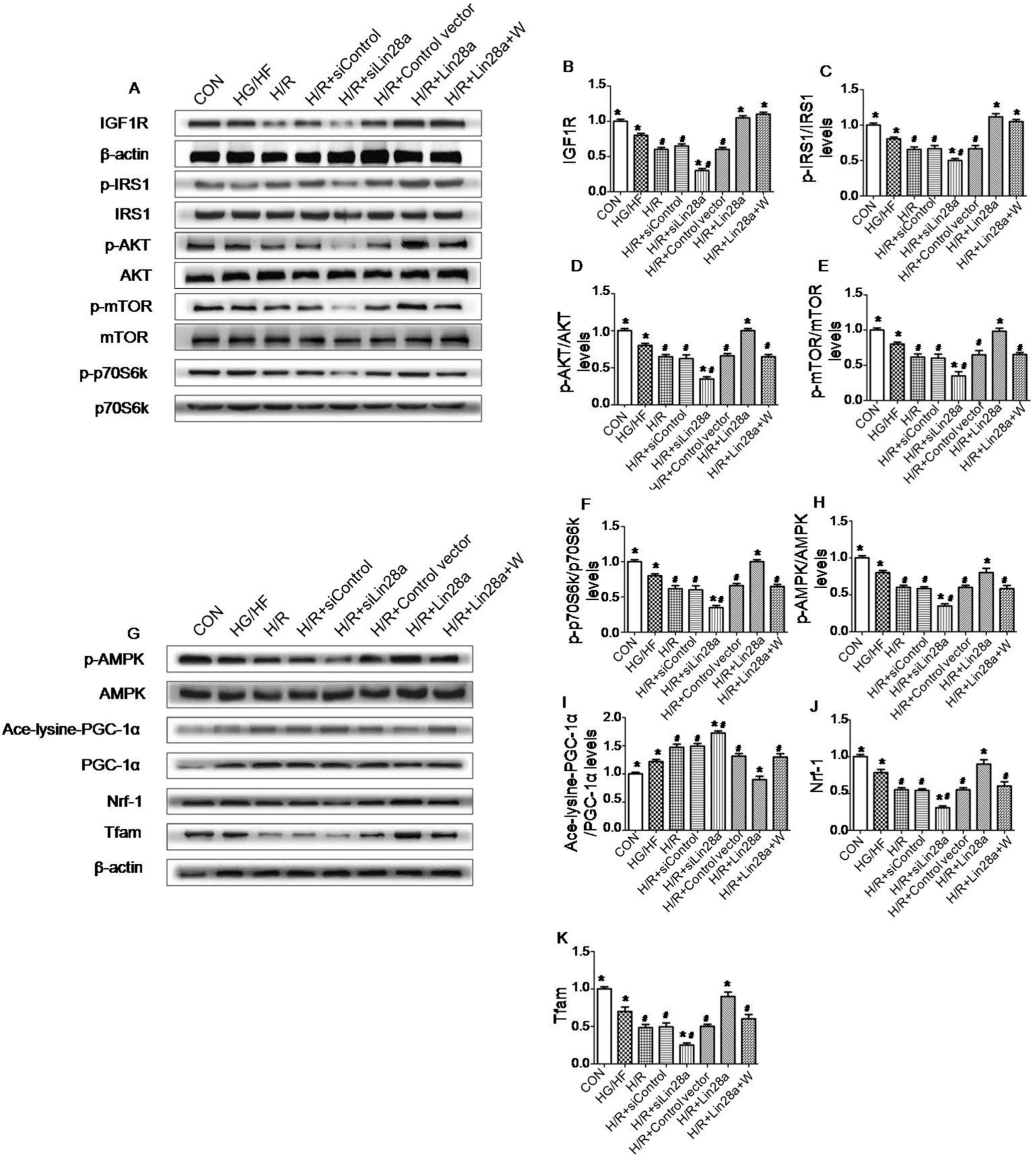
Effect of Lin28a overexpression or knockdown on insulin-PI3K-mTOR signaling pathway and AMPK- PGC-1α signaling pathway activation. Representative gel bolts depicting respective protein expression levels using specific antibodies (A, G); IGF1R (B); phosphorylated IRS-1 (p-IRS-1) (C); phosphorylated Akt (p-Akt) (D); phosphorylated mTOR (p-mTOR) (E); phosphorylated p70s6k (p-p70s6k) (F); phosphorylated AMPK (p-AMPK) (H); Ace-lysine-PGC-1α (I); Nrf-1(J); Tfam(K). The columns and error bars represent means and SD. Data were obtained from at least three independent experiments. *P<0.05 vs. H/R, ^#^P<0.05 vs. H/R+Lin28a.

After 2 h of reoxygenation, western blot analysis revealed that Lin28a overexpression was associated with a significant increase in IGF1R, Nrf-1, Tfam and phosphorylation of IRS-1, Akt, mTOR, p70s6k, AMPK protein levels while a significant decrease in Ace-lysine-PGC-1α protein levels in cardiomyocytes that were exposed to H/R injury in HG/HF incubation (Figure 4). Lin28a siRNA administration decreased IGF1R, Nrf-1, Tfam and p-IRS-1, p-Akt, p-mTOR, p-p70s6k, p-AMPK protein levels while increased Ace-lysine-PGC-1α protein levels in cardiomyocytes underwent H/R injury in HG/HF incubation (Figure 4). Wortmannin (a specific PI3K inhibitor) inhibits p-Akt, p-mTOR, p-p70s6k, p-AMPK, Nrf-1 and Tfam proteins expression while increased Ace-lysine-PGC-1α protein levels as indicated in Figure 4. Thus, the protective effect of Lin28a overexpression on cardiomyocytes was significantly abolished by wortmannin pretreatment.

### Lin28a alleviates cardiomyocytes biomarker release and cytokines levels after H/R Injury under HG/HF conditions

**Figure 5:**
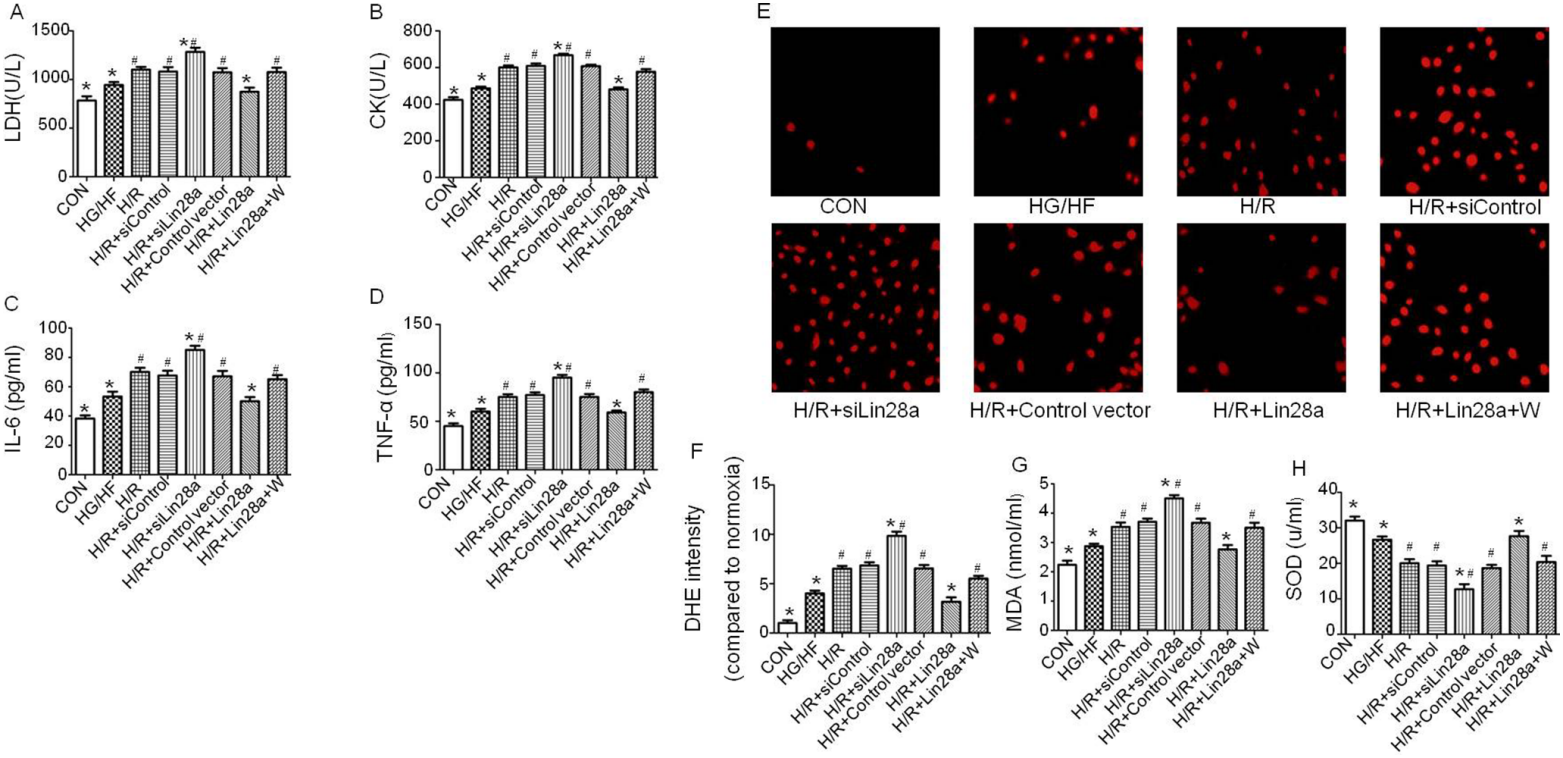
The levels of LDH, CK, IL-6, TNF-α, ROS, MDA and SOD. The LDH, CK, IL-6 and TNF-α levels(A–D); Representative images of DHE staining(E); Quantification of DHE staining(F); MDA and SOD levels(G, H). The columns and error bars represent means and SD. Data were obtained from at least three independent experiments.*P<0.05 vs. H/R, ^#^P<0.05 vs. H/R+Lin28a.

Under HG/HF conditions, the LDH and CK release, IL-6 and TNF-α levels were increased in the cells exposed to H/R injury as compared to the cells without H/R treatment (Figure 5A–5D). Interestingly, under HG/HF conditions, Lin28a overexpression reduced, while Lin28a siRNA administration increased the levels of LDH, CK, IL-6 and TNF-α as compared to the cells undergoing H/R injury alone (Figure 5A–5D). Additionally, inhibition of PI3K with wortmannin abolished the cytoprotective effects of Lin28a on release of LDH, CK, IL-6 and TNF-α (Figure 5A–5D).

### Lin28a decreases H/R induced ROS overproduction, reduces MDA levels and increases SOD levels in cardiomyocytes under HG/HF incubation

Fig. 5E–5H revealed that the ROS and MDA levels were significantly increased while the SOD level was significantly decreased in the HG/HF group compared with those in the control group. Under HG/HF conditions, the levels of ROS and MDA were increased while the SOD level was significantly decreased in the cells exposed to H/R injury as compared to the cells without treatment. Furthermore, under HG/HF conditions, Lin28aoverexpression reduced, while Lin28a siRNA administration increased the H/R injury-enhanced levels of ROS and MDA as compared to the cells undergoing H/R injury alone. Meanwhile, under HG/HF conditions, Lin28a overexpression increased, while Lin28a siRNA administration reduced the H/R injury-decreased levels of SOD as compared to the cells undergoing H/R injury alone. Additionally, inhibition of PI3K with wortmannin abolished the cytoprotective property of Lin28a on the production of ROS, MDA and SOD (Figure 5E–5H).

## Discussion

The increased prevalence of DM is a major concern for health care providers since diabetes incidence is globally increasing and though considered as an epidemic [24]. The National Cholesterol Education Program considers diabetes as a coronary heart disease risk equivalent since diabetic patients without a previous history of myocardial infarction have the same risk of coronary artery disease (CADs) as non-diabetic subjects with a history of myocardial infarction [25], [26]. Actually, diabetes is associated with increased adverse outcomes in terms of both morbidity and mortality over the short and long term after a myocardial ischemic event. Diabetic patients had two to four times higher rates of mortality due to heart disease. The adverse prognosis may be at least in part because of an increase in the myocardial injury in response to ischemia and reperfusion.

Lin28 was first characterized in the nematode Caenorhabditis elegans as a regulator of developmental timing. Mammalian Lin28 exists as two highly conserved paralogs, Lin28a and Lin28b, both of which bind to the terminal loops of the precursors of let-7 family miRNAs and block their processing into mature miRNAs [27], [28], [29]. Recently, Lin28a has been found to be related to insulin resistance and glucose uptake [12]. Lin28a regulates growth, glucose tolerance, and insulin sensitivity in an mTOR-dependent manner in vivo [12]. Meanwhile, Lin28a promotes tissue repair at least in part by enhancing oxidative metabolism and bioenergetics. Mechanistically, enhanced ATP/AMP and GTP/GMP ratios could supply the higher energetic needs of anabolic biosynthesis, mitosis, and migration during tissue repair or could promote growth-signaling pathways like mTOR, all of which are enhanced by Lin28a [30]. The present study was to investigate whether Lin28a was involved in the regulation of cardiomyocyte H/R injury in high glucose/high fat conditions.

Cardiomyocytes apoptosis, the major pathogenic mechanisms underlying cardiomyocytes H/R injury, was observed by TUNEL staining and flow cytometry analysis. When the apoptotic process is activated, the proapoptotic proteins (i.e., Cleaved Caspase-3 and Caspase-3) levels are upregulated. The Bcl-2 family is a key regulator of physiological and pathological apoptosis. The relative ratio of proapoptotic proteins (i.e., Bax) to antiapoptotic proteins (i.e., Bcl-2) plays a key role in determining cell survival or death. It has been demonstrated that the high ratio of Bax/Bcl-2 is associated with greater vulnerability to apoptotic activation [31]. In the present study, Lin28a overexpression inhibited cardiomyocytes apoptosis after H/R injury in HG/HF incubation as evidenced by TUNEL staining, flow cytometry analysis and decreased cleaved caspase-3, caspase-3 expression levels. On the contrary, Lin28a siRNA administration increased cardiomyocytes apoptosis, exacerbated cardiomyocytes H/R injury in HG/HF incubation.

Previous study have demonstrated that Lin 28a/let7 axis regulate glucose metabolism in part through the insulin-PI3K-mTOR pathway [12], [30]. The insulin-PI3K-mTOR pathway is well known to be involved in the cardioprotection against myocardial ischemia/reperfusion injury [32]. We then investigated whether the effects of Lin28a in protecting against cardiomyocyte H/R injury in HG/HF condition was related to insulin-PI3K-mTOR pathway. The results showed that Lin28a overexpression increased, while Lin28a knockdown decreased IGF1R, p-IRS-1, p-Akt, p-mTOR and p-p70s6k expression levels after cardiomyocytes H/R injury in HG/HF incubation. Interestingly, pretreatment with wortmannin abolished the effects of Lin28a overexpression as evidenced by increased LDH and CK activity, caspase-3 expression levels and increased production of IL-6 and TNF-α, enhanced cardiomyocytes apoptosis and decreased p-Akt, p-mTOR and p-p70s6k expression levels. These results suggest that Lin28a overexpression induces cardioprotective effects through the activation of insulin-PI3K-mTOR pathway.

The function of cardiomyocytes is closely related to mitochondria biogenesis and mitochondrial dysfunction is the primary cause of H/R-induced apoptosis of cardiomyocytes [33]. Modulating mitochondrial function may improve insulin resistance and reduce subsequent cardiac mortality [34], [35]. In the present study, Lin28a overexpression alleviated mitochondria ultrastructure impairment and this effect was abolished by wortmannin pretreatment. Lin28a overexpression increased, while Lin28a siRNA administration decreased the levels of ATP content, CS activity, and complexes I/II/III/IV/V activities compared with the H/R group. Furthermore, the effect of Lin28a on the levels of ATP content, CS activity, and complexes I/II/III/IV/V activities was abolished by wortmannin pretreatment. These results indicated that Lin28a protected against cardiomyocytes H/R injury in HG/HF incubation at least in part by alleviating mitochondrial dysfunction.

Mitochondrial biogenesis is regulated by AMPK related pathway. AMPK activation promotes mitochondrial biogenesis through PGC1-α [36], [37]. PGC-1a is one of the master regulators of mitochondrial biogenesis and oxidative phosphorylation gene expression [38]. Acetylation of PGC-1α negatively regulates its activity then reducing mitochondrial biogenesis. Consistently, Lin28a overexpression increased the expression levels of p-AMPK, Nrf-1 and Tfam. The expression level of Ace-lysine-PGC-1α was decreased in the Lin28a overexpression group. Lin28a siRNA administration decreased p-AMPK, Nrf-1 and Tfam expression levels, increased Ace-lysine-PGC-1α expression levels. Furthermore, the above effects of Lin28a were abolished by wortmannin pretreatment, suggesting that Lin28a may regulate mitochondrial biogenesis by AMPK/PGC-1α pathway.

Diabetes is associated with intracellular and mitochondrial metabolic changes that can result in oxidative stress, characterized by overexpression of cytokines produced by adipose tissue, activated macrophages and other cells. The presence of these inflammatory mediators may cause endothelial dysfunction, an important cause of sudden death among subjects with diabetes. Several cytokines described to be related with diabetes are also involved with the development of cardiovascular disease. TNF- and ROS may activate inflammatory pathways and promote the expression of numerous genes involved in insulin resistance [39], [40], [41]. In this study, we found that Lin28a overexpression inhibited inflammatory responses by modulating inflammatory factors production including IL-6 and TNF-α after cardiomyocytes H/R injury in HG/HF incubation. Intracellular MDA, SOD levels and ROS production could be attenuated by Lin28a overexpression and increased by Lin28a siRNA administration. Furthermore, inhibition of PI3K with wortmannin abolished the cytoprotective property of Lin28a on release of MDA, SOD and ROS production.

In conclusion, Lin28a overexpression reduced cardiomyocyte apoptotic index, improved mitochondria biogenesis, increased ATP production and reduced ROS production as compared with the H/R group in HG/HF conditions. This is accompanied decreased inflammatory factors expression levels. The mechanism responsible for the effects of Lin28a overexpression is mediated, at least in part, by the PI3K/Akt and AMPK/PGC-1α dependent pathway.

## Supporting Information

Table S1 **CTs Values of Lin28a and Let7a expression.**
(DOCX)
