# Supplementary material for: Lin28a Protects against Hypoxia/Reoxygenation Induced Cardiomyocytes Apoptosis by Alleviating Mitochondrial Dysfunction under High Glucose/High Fat Conditions
Source: PLoS One. 2014 Oct 14;9(10):e110580. doi: 10.1371/journal.pone.0110580 (PMC4196990; doi:10.1371/journal.pone.0110580)
Supplement: Table S1 — CTs Values of Lin28a and Let7a expression. (DOCX) [file pone.0110580.s001.docx]

**Supporting Information**

Table S1. CTs Values of Lin28a and Let7a expression

| CTs | Lin28a | GAPDH | Let7a | U6 |
| --- | --- | --- | --- | --- |
| CON | 38.3(0.1) | 34.3 (0.2) | 28.3(0.2) | 24.2(0.1) |
| HG/HF | 41.8(0.2) | 37.3(0.1) | 27.0(0.3) | 23.5(0.2) |
| H/R | 42.7(0.2) | 37.5(0.1) | 26.3(0.4) | 23.2(0.3) |
| H/R+siControl | 39.8(0.2) | 34.7(0.3) | 27.7(0.4) | 24.6(0.3) |
| H/R+siLin28a | 40.8(0.3) | 34.3(0.3) | 26.9(0.1) | 24.3(0.2) |
| H/R+Control vector | 39.5(0.2) | 34.4(0.1) | 27.6(0.3) | 24.5(0.4) |
| H/R+Lin28a | 36.8(0.1) | 33.0(0.2) | 29.0(0.3) | 24.6(0.3) |
| H/R+Lin28a+W | 35.9(0.2) | 32.2(0.1) | 28.4(0.1) | 24.0(0.2) |

Values are presented as mean (SD).
